# Drug hypersensitivity to vancomycin confirmed by basophil activation test: case report

**DOI:** 10.11604/pamj.2025.50.88.46204

**Published:** 2025-04-01

**Authors:** Zakaria Zidane, Chaimaa Chahine, Sanaa Souat, Khadija El Azhary, Karima Mohtadi, Rachid Saïle, Claude Lambert, Abdallah Badou, Ibtihal Benhsaien, Hanane Salih Alj

**Affiliations:** 1 Laboratory of Biology and Health, Research Center of Biotechnologies and Health, University Hassan II of Casablanca, Faculty of Sciences Ben Msik, Avenue Cdt Driss El Harti, B.P 7955, Sidi Othmane-Casablanca, Morocco,; 2 Laboratory of Immunogenetics and Human Pathology, University Hassan II of Casablanca, Faculty of Medicine and Pharmacy, 19 Rue Tarik Ibnou Ziad, B.P. 9154, Casablanca, Morocco,; 3 Laboratory of Immunology, CNRS UMR5307 Labo Georges Friedel, Biology-Pathology Center, *Centre Hospitalier Universitaire* St Etienne, Saint-Priest-en-Jarez, France,; 4 Laboratory of Clinical Immunology, Inflammation and Allergies, University Hassan II of Casablanca, Faculty of Medicine and Pharmacy, 19 Rue Tarik Ibnou Ziad, B.P. 9154, Casablanca, Morocco

**Keywords:** Drug hypersensitivity, vancomycin allergy, basophil activation test, immediate hypersensitivity, case report

## Abstract

Drug hypersensitivity reactions to essential antibiotics like vancomycin pose significant diagnostic and therapeutic challenges, particularly in vulnerable populations such as pediatric patients with immune deficiencies. We present the case of a 7-year-old girl with primary immunodeficiency who experienced immediate hypersensitivity reactions to vancomycin, including urticaria and angioedema, managed with corticoids and antihistamines. The Basophil Activation Test (BAT) conducted two years after the last allergic episode revealed significant basophil activation across all tested vancomycin dilutions, with CD63 and CD203c expression exceeding negative and positive controls. These findings confirm vancomycin hypersensitivity and underscore the BAT's utility as a reliable in vitro diagnostic tool, especially in settings where skin testing or specific IgE assays are unavailable. This case highlights the BAT's potential for broader adoption in clinical allergy practice, particularly in resource-limited environments. It emphasizes the importance of reliable diagnostic methods for managing drug hypersensitivity in high-risk patients.

## Introduction

Drug hypersensitivity reactions to essential antibiotics like vancomycin pose a significant clinical challenge, particularly due to their potential severity, such as angioedema and anaphylactoid reactions, and the need for precise, safe diagnostic approaches [1,2]. These reactions are especially challenging in Morocco, where diagnostic capacities are limited by the lack of standardization and implementation of BAT and the unavailability of specific IgE assays for many drug families, including antibiotics [3]. This underscores the urgent need for enhanced diagnostic resources and greater awareness among healthcare professionals.

The BAT has proven to be a valuable tool in detecting hypersensitivity mechanisms, with sensitivity and specificity demonstrated in reactions to antibiotics, including vancomycin [4-8]. Furthermore, its role in monitoring therapeutic responses and desensitization has been highlighted by many studies. This case report discusses a pediatric patient with severe vancomycin hypersensitivity, emphasizing the clinical manifestations, BAT results, and findings, contributing to the growing evidence supporting the BAT as an innovative diagnostic approach.

## Patient and observation

**Patient information:** this case involves a 7-year-old girl with an undocumented primary immunodeficiency who presented with a history of severe reaction to the Ceftriaxone (2020) and Vancomycin (2022).

**Clinical findings:** in December 2024 during hospitalization for recurrent infection, vancomycin treatment was initiated, immediately developed angioedema and generalized pruritus.

**Timeline of current episode:** November 2022: last reaction to vancomycin. December 2024: patient referral to healthcare unit. December 2024: BAT was performed.

**Figure 1:**
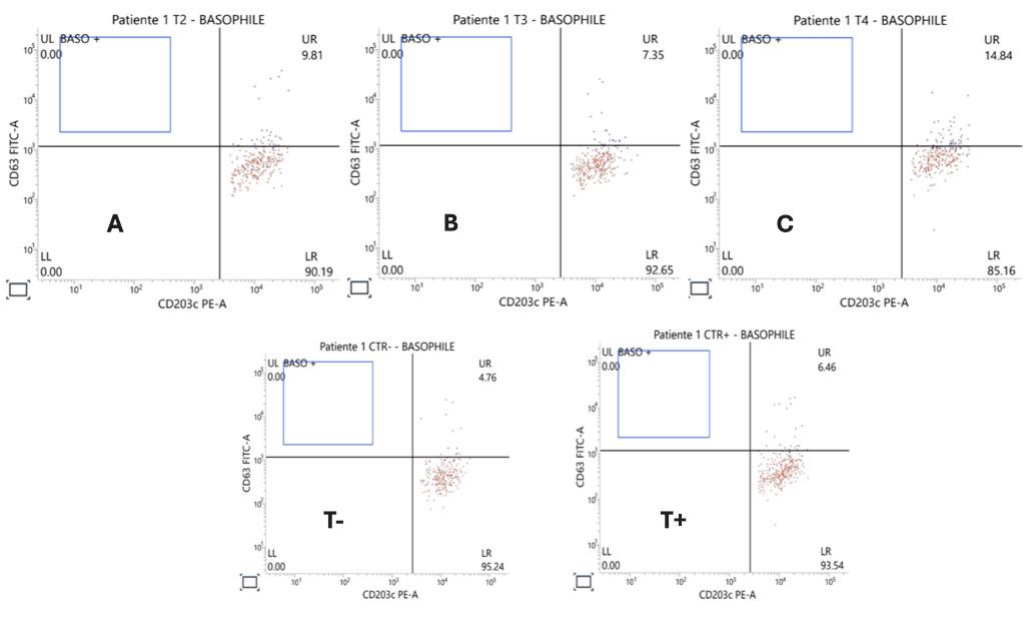
dot plot of BAT with vancomycin: A) activation at 4mg/ml; B) activation at 2mg/ml; C) activation at 1mg/ml; T+) positive control; T-) negative control

**Diagnostic assessment:** the BAT was performed for vancomycin. Results confirmed hypersensitivity to vancomycin in a pediatric patient with primary immunodeficiency. Significant basophil activation was observed at all tested dilutions (1/100 [1mg/ml], 1/50 [2mg/ml], and 1/25 [4mg/ml]), with CD63 and CD203c expression levels of 14.84%, 7.35%, and 9.81%, respectively (Figure 1). These results exceeded the negative control (4.76%) and, at the 1/100 dilution, surpassed the positive control (6.46%). The findings demonstrate a specific and robust basophil response to vancomycin, with an inverted dose-dependent pattern commonly associated with hypersensitivity reactions. This assessment underscores the utility of BAT as a diagnostic tool in cases of drug hypersensitivity, especially when other diagnostic methods, like skin testing or specific IgE assays, are unavailable or contraindicated. In this specific case, the BAT provided reliable confirmation of vancomycin hypersensitivity, aiding in the management and future avoidance of this critical allergen.

**Diagnosis:** the elevated activation markers (CD63 and CD203c) in response to vancomycin across all tested dilutions indicate a clear hypersensitivity reaction. The patient's history of immediate allergic symptoms (urticaria and angioedema) aligns with these findings, reinforcing the diagnosis of vancomycin allergy.

**Therapeutic interventions:** discontinuation of vancomycin and the administration of corticosteroids (60mg/day) and antihistamines (15ml/day).

**Follow-up and outcome of interventions:** allergological diagnosis (prick skin test and specific IgE) were unavailable. A substitute treatment with gentamycin caused no adverse effects and a good resolution of the infection.

**Patient perspective:** patient's guardian: I expect to diagnose if my daughter is allergic to vancomycin and the BAT will show us and help us choose the right alternative, especially since my daughter often uses antibiotics with her recurrent infections.

**Informed consent:** this study was conducted in accordance with the principles outlined in the Helsinki Declaration and obtained approval from the regional ethics committee “Medicine and Pharmacy Faculty of Casablanca/University Hospital Center Bnou Rochd Casablanca” [N° ordre: 11/2022], to ensure that the study complied with ethical standards and protected the rights and well-being of the participants (Law 28-13, N° 02/DRC/00). Before participating in the study, the legal guardians of participant (father and mother) were required to provide informed consent. Confidentiality of data collected was ensured by using identifiers rather than the names of participants.

## Discussion

The results of this study confirmed significant basophil activation at all tested vancomycin dilutions, with the highest response at 1/100 (14.84%), exceeding both the positive (6.46%) and negative (4.76%) controls. These findings align with Chirumbolo *et al*. [5] assertion that CD63 expression is a reliable marker for allergic reactions [1,6]. In this case, the BAT provided definitive evidence of vancomycin hypersensitivity, particularly valuable given the inability to perform skin testing due to clinical constraints. This underscores the utility of the BAT as a safer diagnostic option, particularly for high-risk patients [9].

The strengths of this study include the detailed BAT protocol, which utilized validated markers (CD63 and CD203c) and robust flow cytometry methods to ensure accurate basophil identification. Furthermore, the BAT's non-invasive nature and capacity to confirm hypersensitivity without exposing the patient to allergens demonstrate its clinical applicability in complex cases. Previous studies, such as those by Charpy *et al*. and *et* Suk *et al*. have similarly highlighted the BAT's effectiveness in diagnosing antibiotic hypersensitivity while mitigating risks associated with traditional methods [6,7].

Despite its strengths, this study highlights limitations that must be addressed. The lack of standardized BAT thresholds for vancomycin complicates broader clinical application. Additionally, while the BAT was instrumental in this case, its accessibility remains limited in many healthcare settings, as noted by Petrișor *et al*. [9]. Expanding access and standardizing testing protocols are essential to improve the diagnostic utility of the BAT, particularly for rare hypersensitivity reactions such as those caused by vancomycin.

This case is the first documented instance of a positive BAT result in a patient with vancomycin-induced anaphylaxis, providing a valuable precedent for future research and clinical management. While further studies are needed to refine its application, this case reinforces the BAT's role as a cornerstone diagnostic tool for managing drug allergies, particularly in patients for whom traditional methods are contraindicated or unavailable [9,10].

## Conclusion

This case of a 7-year-old patient with primary immunodeficiency and confirmed vancomycin hypersensitivity, diagnosed through the BAT, underscores the test's critical role in safely and accurately diagnosing drug hypersensitivity in high-risk patients. The study highlights BAT's potential to fill diagnostic gaps when conventional methods are contraindicated, advocating for its integration into clinical practice to improve management of complex allergic reactions.
